# Supplementary material for: Generative design of intrinsically disordered protein regions with IDiom
Source: bioRxiv. 2026 Apr 11:2026.04.10.717777. Preprint. [Version 1] doi: 10.64898/2026.04.10.717777 (PMC13082070; doi:10.64898/2026.04.10.717777)
Supplement: Supplement 1 [file NIHPP2026.04.10.717777v1-supplement-1.pdf]

bioRxiv preprint doi: https://doi.org/10.64898/2026.04.10.717777; this version posted April 11, 2026. The copyright holder for this preprint (which was not certified by peer review) is the author/funder, who has granted bioRxiv a license to display the preprint in perpetuity. It is made available under aCC-BY 4.0 International license.

Supplementary Information

Datasets

Here we describe the datasets provided on HuggingFace:
Datasets (https://huggingface.co/datasets/jxliu2/idiom-datasets)
Below, we describe the data files under idr_datasets/training_sequences:

- AFDB_IDR_90_reps.fasta contains the 53M cluster representatives after the initial 214M full length AFDB protein sequences are clustered at 90% identity, 80% coverage.

- AFDB_IDR_90_alldata.h5 contains 73M IDRs as extracted from the AFDB according to the Tesei logic [32] (see Methods), and after filtering for IDRs belonging to the 53M cluster representatives identified in AFDB_IDR_90_reps.fasta. This HDF5 file contains the following keys: <KeysViewHDF5 ['accession_ids', 'full_avg_plddt', 'full_length', 'full_seq', 'idr_end', 'idr_length', 'idr_plddt', 'idr_start', 'idrs']>.

- AFDB_IDR_90_FIM_512.h5 is created from AFDB_IDR_90_alldata.h5 by filtering out IDRs whose full length sequences are longer than 512 residues. We also find that $\sim 1/3$ of records in AFDB_IDR_90_alldata.h5 are fully low-pLDDT sequences, and we filter out those sequences because we find that they are not representative of intrinsically disordered proteins. We only keep sequences with both low- and high-pLDDT regions. We hypothesize that sequences which are fully low-pLDDT are due to AlphaFold2's poor confidence in sequences which are not similar to those seen during training, rather than because they are fully intrinsically disordered proteins. For the remaining 37M IDRs, we apply the fill-in-the-middle (FIM) transformation as well as IDP data augmentation as mentioned in the Methods, and place those records into AFDB_IDR_90_FIM_512.h5. We note that we represent the <N>, <C>, and <I> tokens with 1, 2, and 3, respectively, in this HDF5 file as well as in the codebase. This is the final file used for the precompute and pre-training steps.

- AFDB_IDR_90_FIM_512_full.fasta contains the 37M full length sequences (in correct order, not FIM-transformed) contained in AFDB_IDR_90_FIM_512.h5. The fasta header contains _IDR_X-Y where X and Y are the 1-indexed indices of the start and end (inclusive) of the intrinsically disordered region.

- AFDB_IDR_90_FIM_512_idrs.fasta contains only the sequences of the 37M intrinsically disordered regions in AFDB_IDR_90_FIM_512_full.fasta, without their surrounding context.

We also provide several datasets of sequences generated by our model under idr_datasets/generated_sequences. All generated sequences are provided in FASTA format along with their corresponding autoregressive model log (pickle format).

- Generated IDPs: 100,000 unprompted intrinsically disordered proteins.

- Generated IDRs: 101,700 intrinsically disordered regions generated using 1,017 DisProt flanking contexts prompts (100 generated IDRs per prompt).

- Generated NPM1 IDRs: 100,000 sequences generated using the NPM1 flanking context as the prompt (UniProt: P06748).

- Generated ProtGPS Sequences: 10,000 IDPs generated from post-trained checkpoints. Post-training was done to optimize ProtGPS localization scores for the four target compartments: chromosome, nucleolus, P-body, and stress granule.

bioRxiv preprint doi: https://doi.org/10.64898/2026.04.10.717777; this version posted April 11, 2026. The copyright holder for this preprint (which was not certified by peer review) is the author/funder, who has granted bioRxiv a license to display the preprint in perpetuity. It is made available under aCC-BY 4.0 International license.

Models

Here we describe the model checkpoints and other files provided on HuggingFace:
Models (https://huggingface.co/jxliu2/idiom)
Below, we describe the directories under `idiom/`:

- `base/` contains the checkpoint of our pre-trained base `IDiom` model, along with its configuration files.
- `post_trained/protgps_reward/` contains the checkpoints of `IDiom` post-trained via reinforcement learning using the ProtGPS reward model, one checkpoint per target compartment. In this paper, we analyzed results for 4 compartments: the nucleolus, stress granules, P-bodies, and chromosomes. However, post-training runs were conducted for all 12 ProtGPS compartments (chromosome, nucleolus, nuclear speckle, nuclear pore complex, P-body, PML body, post-synaptic density, stress granule, Cajal body, RNA granule, cell junction, and transcriptional condensate). We leave analysis of the remaining compartments to future work.
- `protgps/` contains the ProtGPS reward model used during reinforcement learning post-training.
- `data/` contains auxiliary files used during training and inference.

Secondary Structure Metric Analysis

Here we present analysis of secondary structure metrics for training, generated, DisProt, and CATH sequences. Secondary structure was assigned per residue using the dictionary of secondary structure of proteins (DSSP) algorithm as implemented in MDTraj [92]. Secondary structure content was then defined as the sum of the mean α-helical and mean β-sheet fractions across all residues. Fig. S1 shows histograms of the average secondary structure content of 100 randomly chosen sequences from the various training, generated, DisProt, and CATH sets of proteins.

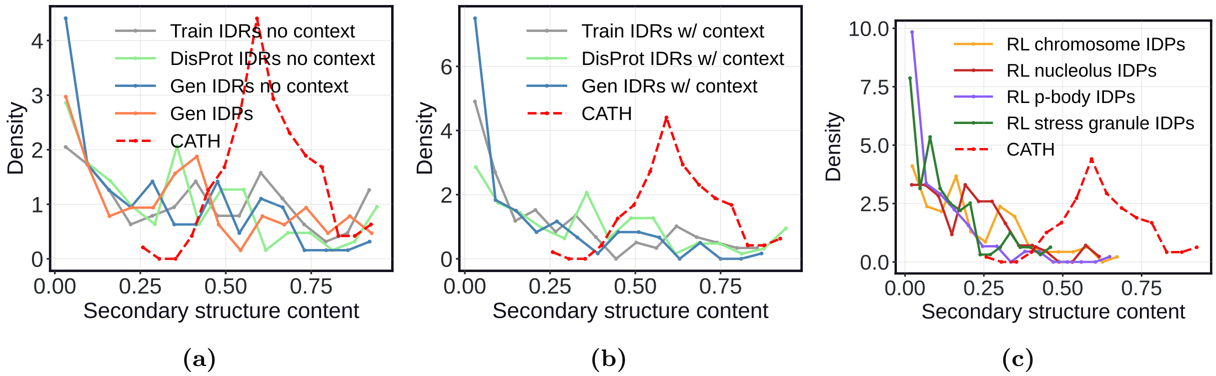

Fig. S1: Secondary structure content analysis. Histograms of of the average secondary structure content ($\alpha + \beta$) for the AF2-predicted structures of 100 randomly chosen sequences from the following sets of sequences: **(a)** Secondary structure content of training IDRs, generated IDRs, DisProt IDRs, and CATH sequences, with their structures predicted with surrounding context included. **(b)** Secondary structure content of training IDPs, generated IDPs, DisProt IDPs, and CATH sequences, with their structures predicted without their surrounding context. **(c)** Secondary structure content of IDPs generated from post-trained `IDiom` checkpoints and CATH sequences, with their structures predicted with surrounding context included.

bioRxiv preprint doi: https://doi.org/10.64898/2026.04.10.717777; this version posted April 11, 2026. The copyright holder for this preprint (which was not certified by peer review) is the author/funder, who has granted bioRxiv a license to display the preprint in perpetuity. It is made available under aCC-BY 4.0 International license.

Disorder Predictions

Here, we present orthogonal disorder predictions from Metapredict v3 [93] and IUPred3 [94]. For both predictors, a higher value represents a higher propensity towards disorder.

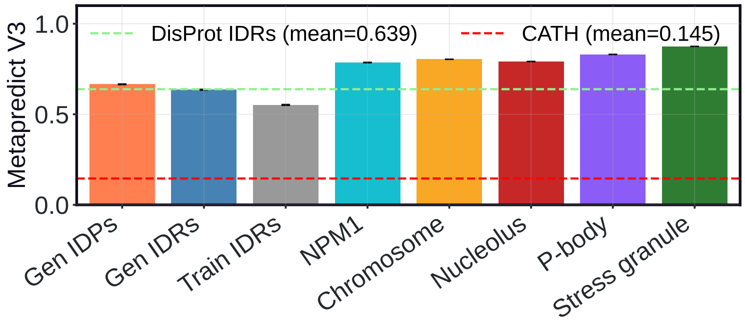

Fig. S2: Disorder predictions from Metapredict V3. Higher values correspond to higher propensity towards disorder. The horizontal green and red dashed lines correspond to the predicted Metapredict V3 values for 1,017 DisProt IDRs and 1,000 CATH sequences, respectively. The bars correspond to predicted Metapredict values for 10,000 sequences generated from `IDiom` for each condition, as well as 10,000 training sequences. The sequences generated from `IDiom` include unprompted IDPs, DisProt-prompted IDRs, NPM1 IDRs, and IDPs generated after post-training for localization to the chromosomes, nucleolus, P-bodies, and stress granules.

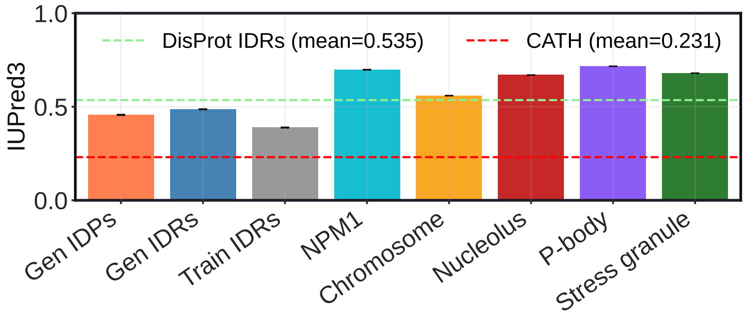

Fig. S3: Disorder predictions from IUPred3. Higher values correspond to higher propensity towards disorder. The horizontal green and red dashed lines correspond to the predicted IUPred3 values for 1,017 DisProt IDRs and 1,000 CATH sequences, respectively. The bars correspond to predicted IUPred3 values for 10,000 sequences generated from `IDiom` for each condition, as well as 10,000 training sequences. The sequences generated from `IDiom` include unprompted IDPs, DisProt-prompted IDRs, NPM1 IDRs, and IDPs generated after post-training for localization to the chromosomes, nucleolus, P-bodies, and stress granules.

ESM3 Comparison

Here we present comparison plots between sequences generated by `IDiom` and `ESM3`, using the same 1,017 DisProt flanking domain prompts. `ESM3` sequences are generated using iterative decoding. A total of 1,000 sequences are sampled for each prompt. As `ESM3` consists of a bidirectional transformer architecture, the length of the generated IDRs is fixed at the length of the ground truth IDR. The number of decoding steps, i.e. forward passes until the sequence is fully unmasked, is set

bioRxiv preprint doi: https://doi.org/10.64898/2026.04.10.717777; this version posted April 11, 2026. The copyright holder for this preprint (which was not certified by peer review) is the author/funder, who has granted bioRxiv a license to display the preprint in perpetuity. It is made available under aCC-BY 4.0 International license.

to be the minimum of 20 and the ground truth IDR length for each prompt. Tokens are sampled with a temperature of 1.0, and all other default inference hyperparameters for ESM3 are used. We find that compared to `IDiom`, `ESM3`-generated IDRs are extremely low-complexity sequences, with a peak in the SEG complexity distribution around 0.5 (Figure S4). We show three example `ESM3`-generated IDRs below, in red:

UniProt P48439:
MNWLFLVSLVFFCGVSTHPALAHFLDLLLLLLLLLLLLLQLILTAILAAIALLLLLLFLL
IVIGILLGLSLGALQLLLLLLLLLLLLSFALQLIFAAILAALLLILLLLLLLIVIGILLS
LSFGALQLLILLLILLLWLLTLLLAKQLKLALALILAAILAALILLLLLLLLLLLLIVIGI
LFGLSLSALQLLLFLLLLLLLLLLVSFALKLKNPISRIIWATLSTFFIICMISAYMFNQI
RNTQLAGVGPKGEVMYFLPNEFQHQFAIETQVMVLIYGTLAALVVVLVKGIQFLRSHLYP
ETKKAYFIDAILASFCALFIYVFFAALTTVFTIKSPAYPFPLLRLSAPFK

UniProt Q9NS23-4:
MPCHPPPLPPPPPPPSPPPEEEEEEEEEIEEEGEEEEPPASPLPPASPPAPEPVEWETPDL
SQAEIEQKIKEYNAQINSNLFMSLNKDGSYTGFIKVQLKLVRPVSVPSSKKPPSLQDARR
GPGRGTSVRRRTSFYLPKDAVKHLHVLSRTRAREVIEALLRKFLVVDDPRKFALFERAER
HGQVYLRKLLDDEQPLRLRLLAGPSDKALSFVLKENDSGEVNWDAFSMPELHNFLRILQR
EEEEHLRQILQKYSYCRQKIQEALHACPLG

UniProt Q14011:
MASDEGKLFVGGLSFDTNEQSLEQVFSKYGQISEVVVVKDRETQRSRGFGFVTFENIDDA
KDAMMAMNGKSVDGRQIRVDQAGKSSDNRGGGGGGGGRGGGGGGGGGGGRGGGGGGRG
GGGSGGGGGGRGGGGGSGGRGGGGGGGGGGGGGGGGGGGGRGGGGGGGGGRY

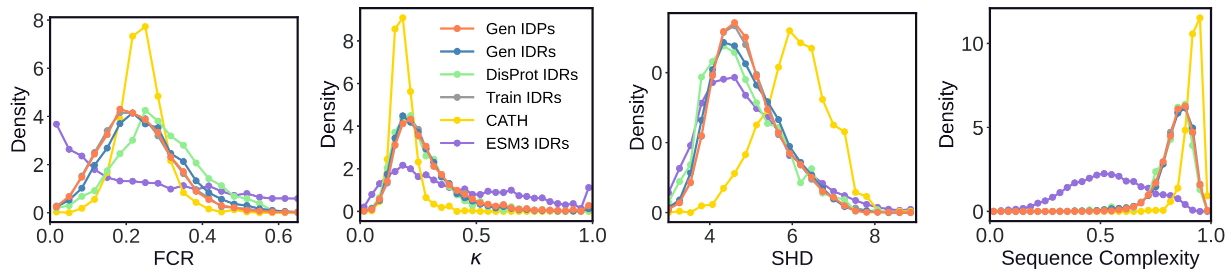

(a) (b) (c) (d)

Fig. S4: Comparison between `ESM3`-generated IDRs and `IDiom`-generated IDRs. **(a)–(d)** Distributions of various sequence metrics for sequences generated from `IDiom` versus `ESM3`. Training set IDRs, natural DisProt IDRs, and folded CATH domains are shown as well. **(a)** Fraction of charged residues (FCR). **(b)** Charge patterning κ parameter. **(c)** Sequence hydropathy decoration (SHD). **(d)** Sequence complexity quantified by the SEG algorithm.

bioRxiv preprint doi: https://doi.org/10.64898/2026.04.10.717777; this version posted April 11, 2026. The copyright holder for this preprint (which was not certified by peer review) is the author/funder, who has granted bioRxiv a license to display the preprint in perpetuity. It is made available under aCC-BY 4.0 International license.

Short Linear Motifs from the Eukaryotic Linear Motif Resource

Here, we list the short linear motifs from the ELM Resource which we scan for, for nuclear localization signals (NLSs) as well as for post-translational modification (PTM) sites (ELM Identifier: MOD).

Nuclear Localization Signals The regular expressions of the 4 NLSs we consider are:

ID	Pattern (regex)
`TRG_NLS_Bipartite_1`	`[KR][KR].{7,15}[DE]((K[RK])|(RK))(([DE][KR])|([KR][DE]))[DE]`
`TRG_NLS_MonoCore_2`	`[DE]((K[RK])|(RK))[KRP][KR][DE]`
`TRG_NLS_MonoExtC_3`	`[DE]((K[RK])|(RK))(([DE][KR])|([KR][DE]))(([PKR])|([DE][DE]))`
`TRG_NLS_MonoExtN_4`	`(([PKR].{0,1}[DE])|([PKR]))((K[RK])|(RK))(([DE][KR])|([KR][DE]))[DE]`

Table 1: ELM NLS motifs and their corresponding regex patterns.

bioRxiv preprint doi: https://doi.org/10.64898/2026.04.10.717777; this version posted April 11, 2026. The copyright holder for this preprint (which was not certified by peer review) is the author/funder, who has granted bioRxiv a license to display the preprint in perpetuity. It is made available under aCC-BY 4.0 International license.

Post Translational Modification Motifs The regular expressions of the 40 PTM MOD sites we consider are:

ID	Pattern (regex)
MOD_AAK1BIKe_LxxQxTG_1	[LIVM][D][DEHYWF]Q.(T)G
MOD_ASX_betaOH_EGF	C.([DN]).{4,4}[FY].C.C
MOD_CAAXbox	(C)[DENQ][LIVMF].$
MOD_CDC14_SPxK_1	(S)P.[KR]
MOD_CDK_SPK_2	...([ST])P[RK]
MOD_CDK_SPxK_1	...([ST])P.[KR]
MOD_CDK_SPxxK_3	...([ST])P..[RK]
MOD_CK1_1	S..([ST])...
MOD_CK2_1	...([ST])..E
MOD_CMANNOS	(W)..W
MOD_Cter_Amidation	(.)G[RK][RK]
MOD_DYRK1A_RPxSP_1	R[PSVA].([ST])P
MOD_GlcNHglycan	[ED]{0,3}.(S)[GA].
MOD_GSK3_1	...([ST])...[ST]
MOD_LATS_1	H.[KR]..([ST])[P]
MOD_LOK_YxT_1	[KR][YF][ÎVEDPGAC](T)[LMIVWFY][RKH]
MOD_NEK2_1	[FLM][PVIED][PVID]([ST])[MLIVF][RKH].
MOD_NEK2_2	[FLMW][P][P]([ST])[PDEGAN][RKH].
MOD_N-GLC_1	.(N)[P][ST]..
MOD_N-GLC_2	(N)[P]C
MOD_NMyristoyl	M{0,1}(G)[ÊDRKHPFYW]..[STAGCN][P]
MOD_OFUCOSY	C.{3,5}([ST])C
MOD_OGLYCOS	C.(S).PC
MOD_PIKK_1	...([ST])Q..
MOD_PK_1	[RK]..(S)[VI]..
MOD_PKA_1	[RK][RK].([ST])[P]..
MOD_PKA_2	.R.([ST])[P]..
MOD_PKB_1	R.R..([ST])[P]..
MOD_Plk_1	.[DNE][PG][ST](([FYILMVW]..)|([PEDGKN][FWYLIVM]).)
MOD_Plk_2-3	[DE]..([ST])[EDILMVFWY](([DE].)|(.[DE]))
MOD_Plk_4	..[ÎRFW]([ST])[ILMVFWY][ILMVFWY].
MOD_PRMT_GGRGG_1	GGRGG
MOD_ProDKin_1	...([ST])P..
MOD_SPalmitoyl_2	G(C)M[GS][CL][KP]C
MOD_SPalmitoyl_4	M{0,1}G(C)..S[AKS]
MOD_SUMO_for_1	[VILMAFP](K).E
MOD_SUMO_rev_2	[SDE].{0,5}[DE].(K).{0,1}[AIFLMPSTV]
MOD_TYR_CSK	[TAD][EA].Q(Y)[QE].[GQA][PEDLS]
MOD_TYR_DYR	..[RKTC][IVL]Y[TQHS](Y)[IL]QSR
MOD_WntLipid	[ETA](C)[QERK]..F...RWNC[ST]

Table 2: ELM MOD motifs and their corresponding regex patterns.

bioRxiv preprint doi: https://doi.org/10.64898/2026.04.10.717777; this version posted April 11, 2026. The copyright holder for this preprint (which was not certified by peer review) is the author/funder, who has granted bioRxiv a license to display the preprint in perpetuity. It is made available under aCC-BY 4.0 International license.

Training Curves

Here, we present additional training curves from pre-training as well as post-training.

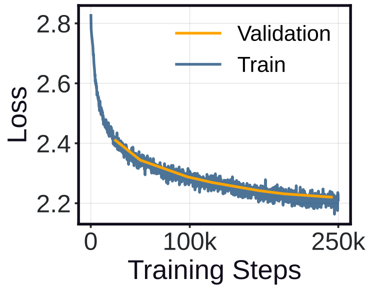

Fig. S5: Pretraining loss curves. Training and validation losses vs optimizer steps during pre-training. The final training loss is 2.19. The final validation loss is 2.22.

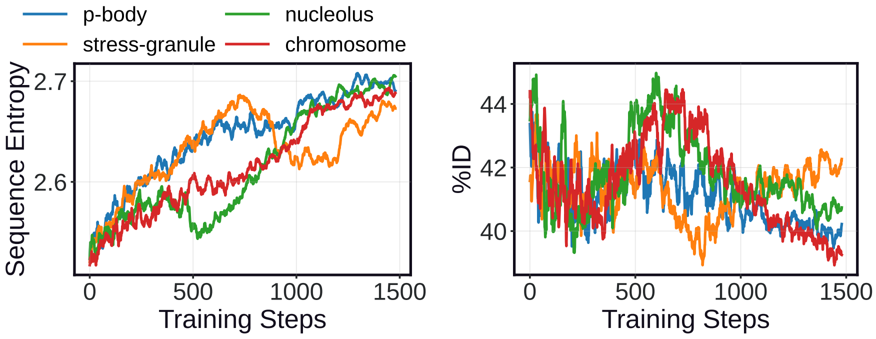

(a) (b)

Fig. S6: Additional post-training curves with the ProtGPS reward model. (a) Shannon entropy vs. training steps (target $H = 2.7$). **(b)** %ID within a generated batch vs training steps (no target value).
